# Silencing of multiple target genes via ingestion of dsRNA and PMRi affects development and survival in *Helicoverpa armigera*

**DOI:** 10.1038/s41598-022-14667-z

**Published:** 2022-06-21

**Authors:** Muhammad Nauman Sharif, Muhammad Shahzad Iqbal, Rukkaya Alam, Mudassar Fareed Awan, Muhammad Tariq, Qurban Ali, Idrees Ahmad Nasir

**Affiliations:** 1 Center of Excellence in Molecular Biology, University of the Punjab, Lahore, Pakistan; 2 Department of Biotechnology, Faculty of Life Sciences, University of Central Punjab, Lahore, Pakistan; 3 Department of Zoology, University of the Punjab, Lahore, Pakistan; 4 University of Management and Technology, Sialkot Campus, Pakistan; 5 Department of Plant Breeding and Genetics, Faculty of Agricultural Sciences, University of the Punjab, Lahore, Pakistan

**Keywords:** Biotechnology, Entomology

## Abstract

RNA interference (RNAi) triggered by exogenous double-stranded RNA (dsRNA) is a powerful tool to knockdown genetic targets crucial for the growth and development of agriculturally important insect pests. *Helicoverpa armigera* is a pest feeding on more than 30 economically important crops worldwide and a major threat. Resistance to insecticides and *Bt* toxins has been gradually increasing in the field. RNAi-mediated knockdown of *H. armigera* genes by producing dsRNAs homologous to genetic targets in bacteria and plants has a high potential for insect management to decrease agricultural loss. The *acetylcholinesterase *(*AChE*),* ecdysone receptor *(*EcR*) and *v-ATPase-A* (*vAA*) genes were selected as genetic targets. Fragments comprising a coding sequence of < 500 bp were cloned into the L4440 vector for dsRNA production in bacteria and in a TRV-VIGS vector in antisense orientation for transient expression of dsRNA in *Solanum tuberosum* leaves. After ingesting bacterial-expressed dsRNA, the mRNA levels of the target genes were significantly reduced, leading to mortality and abnormal development in larva of *H. armigera*. Furthermore, the *S. tuberosum* plants transformed with TRV-VIGS expressing *AChE* exhibited higher mortality > 68% than the control plants 17%, recorded ten days post-feeding and significant resistance in transgenic (transient) plants was observed. Moreover, larval lethality and molting defects were observed in larva fed on potato plants expressing dsRNA specific to *EcR*. Analysis of transcript levels by quantitative RT–PCR revealed that larval mortality was attributable to the knockdown of genetic targets by RNAi. The results demonstrated that down-regulation of *H. armigera* genes involved in ATP hydrolysis, transcriptional stimulation of development genes and neural conduction has aptitude as a bioinsecticide to control *H. armigera* population sizes and therefore decreases crop loss.

## Introduction

The *Helicoverpa armigera* (Noctuidae), is one of the serious herbivorus and cosmopolitan insect pest causing notable economical damage to a broad range of vegetable and field crops^1^. Due to its migratory behavior wider host range, migratory behavior high fecundity, insecticide resistance and multiple generations it is very difficult to control such insect^2^ Numerous control strategies have been proposed or trialed for the control of this pest, including synthetic phytopesticides, microbial pesticides, insecticides, macro-biocontrol agents and transformed crops (e.g. *Bt* cotton)^3^. Most of the insect populations have developed resistance due to improper use of insecticides. Furthermore, development of resistance against number of pesticides in *H. armigera* has been reported globally, including Pakistan. Varied levels of confrontation in *H. armigera* to organo-phosphate and pyrethroid insecticides has been previously described^4^. Nowadays, *H. armigera* is specifically controlled by transformed cotton, encoding *Bt* gene from *Bacillus thuringiensis*. Initially, genetically modified cotton crop was cultivated to eliminate the pesticide resistance from Australia in 1990. Nevertheless, the elevated level of resistance was recorded in existing varieties against *Bt* protein^5^. In China, *Bt* confrontation for *H. armigera* has also been described^6^. It is difficult to manage *H. armigera* pest because of highly polyphagous nature including a wide range of unsupervised non-cultivated plants acting as a determinant of reestablishment and shelters from the insecticide Alternative methods of controlling insect pests are therefore needed.

The knockdown of essential genes through RNA interference (RNAi), has been recently found successful and alternative tool to control damage to our crops by various insects. More than twenty years before RNA interference (RNAi) was reported first time in *Caenorhabditis elegans*^7^ and now this conserved mechanism to suppress the gene expression has been reported in numerous eukaryotes^8^. Subsequently, plant-mediated RNAi technology involving the knockdown of insect genes was developed for managing pests. Several delivery strategies for the use of RNAi in crop protection have been proposed, including spraying, GM plants, virus-induced gene silencing (VIGS) and other in-planta applications^9^. Baum et al.^10^, genetically engineered corn plant to express dsRNA against the *V-ATPase-A* gene of Western Cotton Rootworm to validate the practical use of this approach. The results demonstrated that increased level of insect resistance was observed in transgenic plant than the control plant. Another study reported that reduction in transcript level of a gene *CYP6AE1, which* is generally linked with the gossypol endurance, can enhance the susceptibility of pest to the internal defense mechanism of the plant^11^. Recently, further research has also confirmed the feasibility of the plant-mediated RNAi approach for pest control and showed that it effectively down regulated the transcript level of targeted genes, resulting in larval developmental deformity and mortality^8,12–18^.

In plants, Post Transcriptional Gene Silencing is being used as a functional genomics tool to silence gene expression. In the case of virus-induced gene silencing (VIGS), knockdown is activated by a viral vector expressing dsRNA homologous to the gene of interest, leading to the degradation of both targeted transcripts and the viral genome^19^. Many studies have illustrated the aptitude of transgenic plant-mediated insect management by encoding dsRNA sequences specific to genes crucial for pest perseverance^20^. For instance, transgenic *Nicotiana benthamiana* targeting the *MPC002* gene and *Arabidopsis thaliana* targeting the *Rack1* gene have been demonstrated to critically disturb the aphid natural life span^21^. Knockdown of the *CYP6AE14c* gene causes larva to be more sensitive to gossypol in *Gossypium hirsutum*, and efficient western root worm insect management was accounted for by transgenic *Zea mays* expressing dsRNA targeting the *V-ATPase-A* gene^22^. In the present study we develop a similar plant-virus based dsRNA producing system (VDPS) using VIGS vector for the knockdown of *H. armigera* genes. These findings strongly suggest that plant-mediated RNAi is emerging as a powerful approach for controlling insect pests. The studies also show that genes encoding proteins with essential functions in insects are the best RNAi targets for increasing morbidity and mortality.

To investigate insecticidal activity, three genes engaged in signaling pathways crucial for *H. armigera* progression and neural conduction were targeted. We hypothesized that intervention with these critical pathways would be mortal. *Acetylcholinesterase* (*AChE*) hydrolyzes the acetylcholine neurotransmitter into its acetate and acetyl-CoA, thereby eliminating remaining neurotransmitter molecules from the synaptic cleft that determine regular behavior. *AChE* obstruction provokes elevated acetylcholine concentrations, causing insistent stimulation of glands and muscle and resulting in paralysis, muscle dysfunction and mortality^23^. *Vacuolar-type ATPases* accounted for several ions conveying in insect epithelia. It is a multi-subunit enzyme comprising V1 and V0 domains. Subunit A is the catalytic site of the V1 domain involved in hydrolysis of ATP^24^. The *ecdysone* receptor *EcR* gene, the third target studied in this report, is actively involved in the steroid signaling pathway, whose stimulation persuades a cascade of *ecdysone* receptive genes that are crucial for insect growth. In a recent study, expression of dsRNA to knockdown the *EcR* gene in adult *Drosophila melanogaster* revealed that reduced expression of *EcR* can disturb stress resistance, courtship behavior and reproduction, in addition to distressing sex-mediated signaling and survival in adults^25^.

Findings of this study revealed that both ingestion of bacterially expressed dsRNA coated diet and transgenic potato plant encoding dsRNA specific to the *EcR, v-ATPase-A* and *AchE* genes of *H. armigera* induced mortality and developmental deformity in *H. armigera* larvae.

## Materials and methods

### Insect rearing

*H. armigera* larvae were collected from different geographical regions of Punjab, Pakistan, and maintained at the Entomology Lab, Centre of Excellence in Molecular Biology, University of The Punjab. The sampling of insects (*Helicoverpa armigera*) and plants (potato plant) were carried out according to the relevant guidelines and legislation with appropriate permissions from authorities of the Entomology and Seed Biotechnology Labs respectively, Centre of Excellence in Molecular Biology, University of The Punjab, Lahore Pakistan. *H. armigera* were reared on artificial diet, and adults were maintained in rearing cages (30 × 20 × 20 cm) and fed 10% (honey/water) honey solution. The eggs were collected each successive day and placed on an artificial diet at 26 °C and 60% relative humidity and allowed to hatch.

### Total RNA isolation and cDNA synthesis

Total RNA isolation from the collected samples of 3rd instar larva was carried out using TRIzol reagent (Life Technologies cat no. 15596026). The homogenate of larval tissue samples was made with 1 mL of TRIzol reagent and incubated at room temperature (RT) for 5 min. Two hundred microliters of chloroform was added, and the content was mixed vigorously for 15 s and incubated at room temperature for 10 min followed by centrifugation for 5 min at 13,000 rpm (at 4 °C). The upper clear phase was transferred to another nuclease-free 1.5 mL Eppendorf tube, and 500 μL of prechilled isopropanol was added and incubated for 10 min at room temperature followed by centrifugation. Washing of the pellet was carried out using 500 μL of 75% ethanol, centrifuged again at 13,000 rpm (at 4 °C) for 15 min (2×), and resuspended in 20 μL DEPC water. The cDNA was synthesized using 1 μg of total RNA template from each *H. armigera* larval sample using a Revert Aid First-Strand cDNA synthesis kit (Thermo Fisher Scientific, cat no. 1622) following the suggested practice.

### Amplification of targeted genes

Amplification of the targeted genes was carried out using gene-specific primers (S1) and DreamTaq Green PCR Master mix 2× (Thermo Fisher Scientific, cat no. K1081). The PCR contained 10 μL of DreamTaq Green PCR master mix, 0.8 μL of each template cDNA and 0.4 μL of both forward and reverse primers (10 μmol) (S1) added to a PCR tube (0.2 mL) and nuclease-free water to a final volume of 20 μL. The PCR product was electrophoresed on a 1.2% agarose gel and sequenced. The reverse compliment of the obtained nucleotide sequence of target genes was analyzed using RNAi Designing Tool (BLOCK iT™ RNAI Designer) to select the best region. The most efficient gene fragment edited to replace A from ATG (start codon) with G to prevent the start of translation. The 491 bp, 475 bp, 479 bp and 379 bp gene fragments by g Blocks^®^ Gene Fragments, IDT (S1) of *v-ATPase-A, AChE, EcR* and *egfp* were synthesized respectively.

### Plasmid assembly and dsRNA expression

For the design of the L4440 plasmid to express dsRNA, g Blocks^®^ Gene Fragments of *v-ATPase-A, EcR* and *AChE* genes were amplified by PCR harnessing specific primers. The sequence encompasses *Sma*1 and *Hind*III sites to enable cloning into the multiple cloning site (MCS) of the L4440 vector, and ‘egfp’ is employed as a control. The L4440 plasmid was obtained from AddGene (Plasmid # 1654) and contains two promoters with an inverted orientation flanked by MCS. Verification of constructs including *L4440-AChE*, *L4440-v-ATPase-A* and *L4440-egfp* was performed by PCR, a restriction method and sequencing. Competent cells of HT115 DE3 strain of *E. coli* bacteria that lacked RNase III were developed to treat CaCl_2_, and later, transformation was performed with the genetically engineered plasmid. Then, one colony was chosen from the agar plate and cultured in LB at 37 °C overnight with shaking at 220 rpm. Dilution of culture was performed up to 100 times in 800 mL of 2 × YT accompanied by 75 µg/mL and 12.5 µg/mL ampicillin and tetracycline, respectively, at 37 °C with an optical density OD_600_ = 0.5. T7 polymerase was induced by 0.4 mM IPTG, and additional incubation with transformed *HT115 DE3* strain of *Escherichia Coli* bacteria was performed at 37 °C for 4 h.

### Quantification and purification of dsRNA

Total nucleic acid was extracted as illustrated by Fire et al*.*^26^. The culture of bacteria was centrifuged at 5000*g* for 10 min. The bacterial pellet was resuspended in I M acetate or 10 M EDTA supplemented with the exact volume of phenol:chloroform:isoamyl alcohol (25:24:1). Then, the samples were incubated at 37 °C and centrifuged at 12,000*g* for 15 min. The upper transparent layer was mixed with isopropanol and kept at − 20 °C overnight. Next, the samples were centrifuged at 12,000*g* for 30 min. Samples were mixed with RQ1 RNase-free DNase to eliminate ssDNA and then with RNase to remove ssRNA. The dsRNA pellet was resuspended in Tris–EDTA solution (1X) pH 7.5. Resuspended dsRNA was loaded onto a 1.2% agarose gel treated with ethidium bromide and visualized under UV light. The concentration was measured with a NanoDrop 1000 (Thermo Scientific).

### Persistence of dsRNA in artificial diet and gut

The evaluation of dsRNA in the gut was determined as desribed by *Vatanparast and Kim*^27^. The gut juice was isolated from L1, L2, L3, L4 and L5 instar larvae of 4, 7, 10, 13 and 18 days old larvae respectively by collecting supernatant of gut content followed by centrifugation at 12,000×*g* for 5 min at 4 °C. 10 mL gut juice of each sample was mixed with 5 µg of dsRNA and incubated at room temperature for 1 h. The stability of dsRNA throughout the feeding process was evaluated on a 1.2% agarose gel TAE. Extraction of dsRNA from the diet was also performed and evaluated on a 1.2% agarose TAE gel.

### Feeding bioassays on artificial diet coated with dsRNA

In-vivo feeding assays were performed with newborn larva. Newly hatched larvae of *H. armigera* were transferred into a 30 cm × 30 cm × 30 cm box containing an artificial diet coated with dsRNA. Thirty larvae were selected for each analysis, and the test was repeated 3 times. Larvae were starved for 4 h prior to in-vivo feeding experiments to maximize the consumption of diet. The synthetic diet was prepared and cut into small pieces of 3 cm^2^. Each exposed side of the diet was coated with 100 µL of dsRNA solution. Pieces of artificial diet containing dsRNA were replaced twice a day. *H. armigera* was constantly nourished on the diet for 10 days and then maintained on an artificial diet without dsRNA to evaluate post feeding effects. Collection of individuals was preceded on days 2, 4, 6, 8 and 10 to conduct qRT-PCR. However, before qRT-PCR, individuals were kept in liquid nitrogen and stored at − 80 °C. TRIzol reagent (Life Technologies cat no. 15596026) was used to extract RNA from larva, and the death rate was noted on each successive day up to day 10.

### VDPS mediated gene silencing

The pTV (TRV2) vector harboring ≥ 450 bp of insect genes was used as a virus-derived dsRNA production system (VDPS). The pTV (TRV2) Virus Induced Gene Silencing (VIGS) vector containing the *EcR *(*pTV-EcR*),* AChE *(*pTV-AChE*) and *vAA *(*pTV-vAA*) gene fragments in antisense orientation was transformed into GV3101 strain of *Agrobacterium tumefaciens* and TRV1 in C58C1 *A. tumefaciens* strain respectively. Positive colonies were confirmed by PCR and restriction digestion and inoculated into 25 mL *YEP* medium with 20 mg mL^−1^ rifampicin and 50 mg mL^−1^ kanamycin. After 48 h of incubation at 28 °C, cultures were centrifuged and diluted in induction medium containing 10 mM MgCl_2_, 200 μM acetosyringone and N-morpholino ethane sulfonic acid with pH = 5.5 and incubated overnight at room temperature. Infiltration of *Solanum tuberosum* leaves after 28 days of plantation was carried out using TRV1 and TRV2 components of the VIGSS system mixed in an equal ratio (1:1). Potato plants transformed with empty vector (EV) were used as a control. Plants were maintained in an environmentally controlled growth chamber at 23 ± 2 °C and 60–70% relative humidity. The insertion of the transgene was confirmed by PCR using DreamTaq Green PCR master mix 2× (Thermo Fisher Scientific, cat no. K1081). The thermocycler parameters of PCR were 3 min at 94 °C (1 cycle), 30 s at 94 °C, 30 s at 56 °C and 45 s at 72 °C for 35 cycles and a final extension step at 72 °C for 7 min. The primer pairs were designed to amplify 243, 168 and 186 bp of the kanamycin resistance gene in *TRV-vAA-, TRV- EcR-* and *TRV-AChE*-transformed plants, respectively (S1). The seeds of virus-susceptible *Solanum tuberosum* (Desiree variety) used in this study were provided by the Ayub Agriculture Research Institute (ARI), Faisalabad, Pakistan.

### Dot blots analysis

Total RNA was extracted from VDPS-positive plants using TRIzol reagent according to the manufacturer’s protocol (Thermo Fisher Scientific, USA). The concentration and quality of RNA were estimated using a Nanodrop Spectrophotometer. Re-suspension of total RNA samples was carried out by dissolving 20 μg of RNA in 13% MOPS buffer, 66% formamide and 21% formaldehyde followed by denaturation at 65 °C for 15 min. The diluted RNA samples were spotted on a positively charged nylon membrane (Hybond-N+, GE-Amersham, UK) dipped in 6× SSC solution (pH = 7), and Whatman filter paper was used to dry the nylon membrane. RNA spots were fixed and cross linked under UV light at 300 nm for 30 s (TWice). DIG (Dioxigenin-Labeled UTP)-tagged probes of *v-ATPase-A, EcR* and *AChE* were prepared using DIG-high Prime DNA Labeling and Detection Starter Kit I (Roche, Cat no. 11745832910) according to the standard protocol. Spots of RNA were detected using chromogenic techniques after 6 h of incubation in BCIP/NBT solution at 25 °C.

### Bio-assay on VDPS potato plant

In-vivo feeding assays were performed with newborn larva as described previously. For the in-vivo feeding assay on the transformed (transient) plants, larvae were placed on the leaves of transformed and control plants (infected with empty TRV vector) maintained in cages at 23 °C under a 16-h light period and 8-h dark period in a controlled growth chamber. Plants were covered with a polyethene bag to settle down larva on plant leaves. For each experiment, 30 larvae were used and monitored daily by recording mortality, developmental changes and larval weight. In the experiments with transformed or control plants, anti-feeding activity was also monitored by assessing the leaf damage area. In a preliminary experiment, larvae were allowed to feed for 5 days, and the response to 4, 6, 8 and 10 days post-infiltrated plants was evaluated to determine the best time of in-vivo feeding after viral incursion. In another experiment, larvae were allowed to feed for 10 days on 10-day-old VDPS plants, and the experiment was run in three replicates. Larval weight, development and mortality were recorded each day.

### Quantification of gene expression by RT-qPCR

Larvae were collected on days 0, 2, 4, 6, 8, and 10 after their release to feed on VDPS plants and diets coated with dsRNA. TRIzol reagent was used to extract total RNA according to the standard protocol. Extracted RNA was quantified using a Nanodrop, and 1 µg of total RNA was subjected to cDNA synthesis according to the manufacturer’s instructions (First Strand cDNA Synthesis kit Thermo Fisher, USA # K1612). Expression of targeted genes in *H. armigera* larva fed a diet coated with dsRNA and potato plants transiently expressing dsRNA was quantified by RT-qPCR. 20 μL reaction mixture contained 1 µL of each template cDNA and 0.5 μL of both forward and reverse primers (10 pmol) (S1), 10 μL of SYBR^®^ Green PCR Master Mix (2×) and 8 μL of dd H_2_O. 18S ribosomal RNA and β-Actin were used as internal controls.

The thermal profile of the qRT-PCR mixture was 94 °C for 5 min followed by 40 cycles, comprising 94 °C (for 30 s), 60 °C (for 30 s) and 72 °C (for 30 s). RT-qPCR was performed using the PikoReal™ Real-Time PCR System, and each reaction was run in triplicate. Melt curve analysis was performed at the end of each reaction to evaluate the specificity of the amplification product from 60 to 85 °C with an increase of 0.5 °C every 10 s. The 2^−ΔΔCT^ approach was used to analyze quantification results^27^. Standardization of consequent CT values was carried out using β-Actin and 18S ribosomal RNA as reference genes. The gene expression level was calculated in different larval groups, and the fold variation in the transcript levels of targeted genes was determined as described previously.

### Statistical analysis

The data collected from the abovementioned experimental replicates were analyzed by SPSS 17 (IBM Corporation, Somers, NY) for one-way analysis of variance (ANOVA), and Duncan’s test was employed to assess the significant difference between different test groups. p < 0.05 was considered as significant.

## Results

### Cloning of partial CDS of *AChE, EcR* and *v-ATPase-A*

The amplified partial CDSs obtained for the three proteins were 656 bp, 700 bp and 627 bp for *v-ATPase-A AChE* and *EcR,* respectively (GenBank accession numbers MG457281, MG030629, and MG457282). The BLASTN results showed high similarity of approximately 95%, 93% and 96% for *v-ATPase-A AChE* and *EcR,* respectively. The obtained nucleotide sequences were analyzed using the RNAi tool by Thermo Fisher to determine the optimal dsRNA fragment (< 500 bp). The resulting fragments comprising 491 bp, 475 bp and 479 bp of *v-ATPase-A, AChE* and *EcR* (S1) were cloned in antisense orientation into the L4440 dsRNA vector and expressed in *HT115* cells by induction with 0.04 M IPTG. The dsRNA was extracted and purified as prescribed by Fire et al.^26^.

### Stability of dsRNA in diet

**Figure 1:**
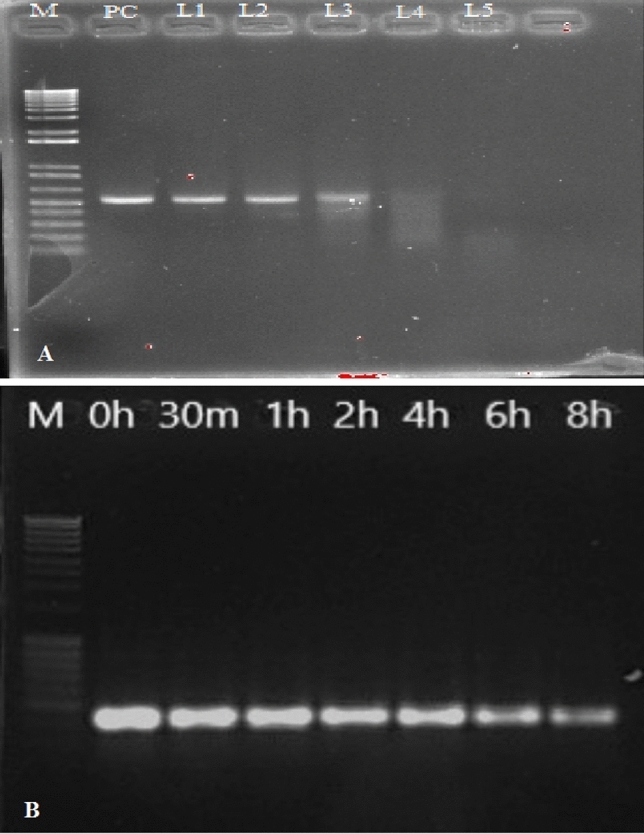
Stability of dsRNA in Gut juice of *H. armigera* and artificial diet. (**A**) Gut juice was collected from the midgut of *H. armigera* larvae by collecting supernatant of gut content followed by centrifugation at 12,000×*g* for 5 min at 4 °C. Effect of gut juice on dsRNA specific to *AchE* after incubating 5 μg dsRNA with 10 μL gut juice sample from each larval stage (L1–L5) for 1 h at room temperature. After incubation, all dsRNA samples were subjected to 1% agarose gel electrophoresis. (**B**) Stability of dsRNA specific to *AchE* in diet was determined by mixing dsRNA with the diet. The dsRNA from artificial diet was extracted and examined by 1% agarose gel electrophoresis for integrity at different intervals of time.

The stability of dsRNA in gut juice was determined to evaluate the RNase activity at different larval stages. The result of the dsRNA stability in gut revealed that gut juice of L4 and L5 show high level of RNase activity as compared to the gut juice of L1- L3 larvae (Fig. 1A). The results of gel electrophoresis demonstrated that dsRNA remained stable in the diet even after 8 h of addition (Fig. 1B). Therefore, we consider that the time of addition and feeding time of *H. armigera* is 9:00 and 15:00, respectively, with a fresh diet containing dsRNA twice a day between 9:00 and 11:00 and between 15:00 and 16:00 to ensure the stability and availability of dsRNA in the diet^28^.

### Optimization of oral delivery of dsRNAs

**Figure 2:**
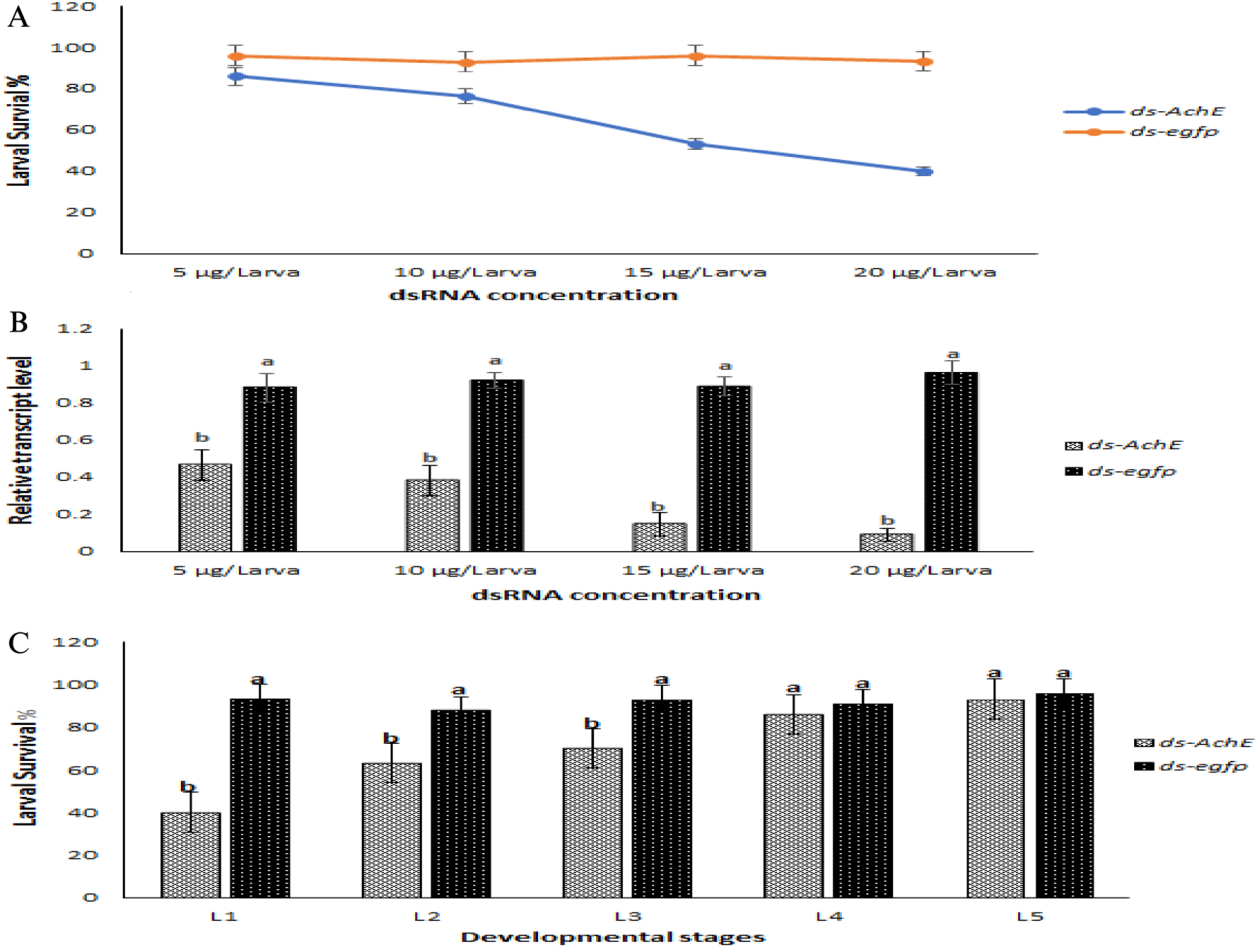
Optimization of dsRNA delivery in *H. armigera.* (**A**) Larval survival rate at different dsRNA concentrations. (**B**) Relative expression of *AChE* transcripts in *H. armigera* L1 larva exposed to different dsRNA concentrations. (**C**) Mortality at different developmental stages (L1–L5) of *H. armigera* larva by ingesting dsRNA-coated diet (20 µg/larva) for 5 days. Significant mortality was observed in L1–L3 stage compared to L4 and L5 larvae. Different letters above standard deviation bars indicate significant difference. For each experiment 30 larvae were used and p < 0.05 was considered as significant.

To determine the optimum dose of dsRNA for maximum larval mortality, newly hatched larvae were fed a diet coated with different concentrations of dsRNA. When larvae were fed a dsRNA-coated diet, the survival rate dropped significantly with the increased dose of dsRNA in all experimental groups. Significant insecticidal activity was observed after 2 days in all treatments compared with the control group. However, the insecticidal activity of *ds-AChE* was much higher than that of the control group. The survival rate of *ds-AChE* dropped even at 10 µg/larva (67%), and a further decline was observed at 15 µg/larva (53%) and 20 µg/larva (40%) compared with the control group (Fig. 2a). The treatment also interfered with larval growth and development, resulting in reduced body weight and size compared to the control group.

To evaluate the ideal larval stage for oral dsRNA delivery, larvae at different larval stages (L1–L5) were fed a diet coated with 20 µg/larval dsRNA. When larvae were fed a dsRNA-coated diet for 5 days, they suffered substantial mortality at the L1, L2 and L3 stages (Fig. 2b). Most of the older larvae survived, but some of them had reduced body weight and size. Significant mortality in L4 and L5 larva was not observed that might be due to the high level of RNase activity as described earlier. Therefore, all of the experiments were conducted using newly hatched larva (Fig. 2c).

### Ingestion of bacterially expressed dsRNA-induced RNAi in *H. armigera*

**Figure 3:**
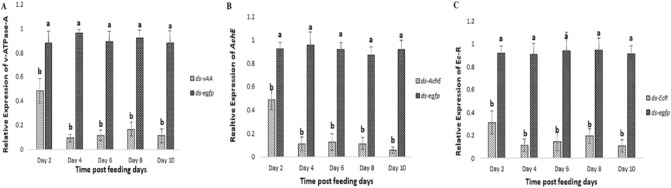
Knockdown of *v-ATPase-A* (**A**),* AChE* (**B**) and *EcR* (**C**) of *H. armigera* larva (L1) by ingestion of dsRNA. Larval samples from each group were collected on different days. *ds-egfp* was used as a control. Different letters above standard deviation bars indicate significant differences. All experiments were triplicated. p < 0.05 was considered as significant.

To evaluate whether the oral uptake of dsRNA induced a specific RNAi knockdown effect, qRT-PCR was performed to quantify the mRNA level of each targeted gene. Each experiment was run in triplicate. The qRT-PCR outcomes revealed that oral delivery of dsRNA induced RNAi in *H. armiger*a. Among the three different groups fed a dsRNA-coated diet, all three genes showed substantial depletion of targeted mRNA transcripts compared to the control group fed *ds-egfp* on certain days (Fig. 3). The maximum depletion of each targeted gene in larva fed a dsRNA-coated diet ranged from 43 to 94%. The lowest level of *v-ATPase-A* transcript was observed on day 4, with a 91% reduction compared to the control *ds-egfp* group (Fig. 3A). *EcR* mRNA showed significant down regulation on both day 4 and day 10 with 89% depletion compared to the control group (Fig. 3C). The larval group treated with *ds-AChE* showed a maximum down regulation of 93% on day 10 (Fig. 3B).

### Ingestion of dsRNA specific to *AChE, v-ATPase-A* and *EcR* possesses insecticidal activity against *H. armigera* larva

**Figure 4:**
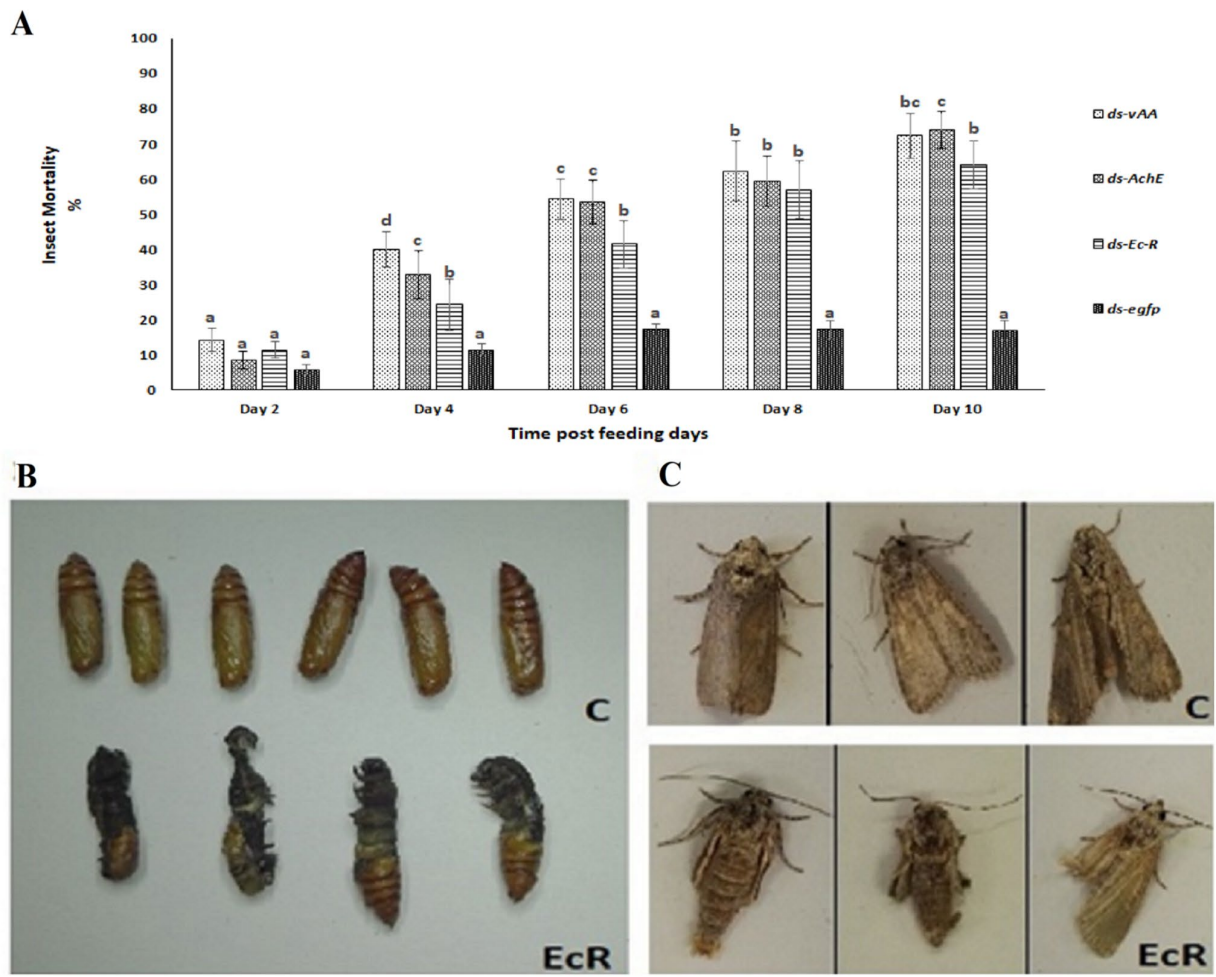
Mortality and abnormal phenotype development in *H. armigera* larva fed on bacterial expressed dsRNA. (**A**) Average mortality of 1st instar *H. armigera* larva fed on *ds-EcR, ds-v-ATPase-A, ds-AChE.* The mortality rate of *H. armigera* 1st instar larva in dsRNA treatments in a 10-day in-vivo feeding bioassay was calculated. Different letters above standard deviation bars indicate significant differences. All experiments were performed in triplicate, and p < 0.05 was considered significant (Duncan’s test). (**B**) Inhibition of pupation in *H. armigera* larva fed on *ds-EcR* or *TRV-EcR potato plants*. Some insects died as larval-pupal intermediates. (**C**) Abnormal adult emergence. *C* Control group fed *ds-egfp* or *TRV-EV* potato plants, *EcR* experimental group fed *ds-EcR* or *TRV-EcR* potato plants.

Our results were different from the data of previous research showing relatively high mortality (Fig. 3A). The maximum mortality rates were observed in the *ds-AChE* and *ds-vAA* treatment groups. *ds-AChE* proved to be lethal to *H. armigera* larva, with ≥ 74% mortality in the in-vivo bioassay, which was significantly higher than that in the *ds-egfp* group (p ≤ 0.05). *EcR* and *v-ATPase-A* dsRNA-coated diets caused 64% and 72% mortality in *H. armigera* larva after 10 days of feeding (Fig. 4A).

### dsRNA specific to *EcR* possesses molt-inhibiting activity against *H. armigera*

It was speculated that oral delivery of dsRNA specific to *EcR* might cause abnormal development in lepidopteran insects. Therefore, larva fed with *ds-EcR* coated diet were maintained on an artificial diet after 10 days of exposure to dsRNA until pupation and adult emergence to evaluate the post feeding effect of dsRNA. Although knockdown of targeted transcripts could be detected by qRT-PCR, a negative effect on growth, development and phenotype was not found by ingesting dsRNA for 10 days except in the case of *ds-EcR* larval groups. The larva fed with the *ds-EcR* coated diet showed slow or no movement, and their development was seized compared to that of the control group.

In addition to mortality, a developmental abnormality at metamorphosis was also recorded in the *ds-EcR* treatment group. For the first 6 days, the physical appearance of larva was normal. However, deformed phenotypes were observed 9–11 days later. In the larval developmental process, some insects moved sluggishly or not at all, and some were unable to complete molting development. This may be because of the old cuticle could not disengage from the larval body and the new cuticle was not fully developed at the moment of eclosion (Fig. 4B). Additionally, regarding pupation, abnormalities were observed in the *ds-EcR* treatment group, where some larva failed to detach the cuticle from the last instar, and some were not developed in the pupa (Fig. 4B). Impaired adults emerged from pupae with smaller body sizes, deformed wings and lower body weights (Fig. 4C).

### Generation and molecular characterization of VDPS plants

**Figure 5:**
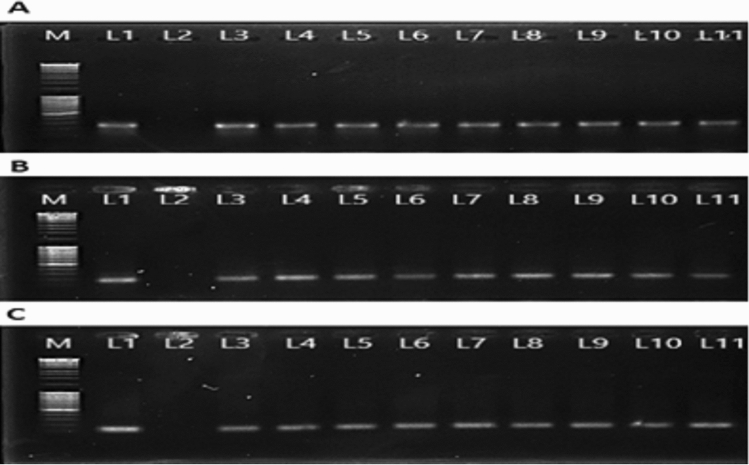
Molecular analysis of VDPS plants. (**A**) Amplification of the kanamycin gene in the TRV-vAA potato lines by PCR amplification of fragment size 251 bp. Loading sequence of the samples in 1.2% agarose gel is, M = 1 kb DNA Marker, Lane 1 = positive control, Lane 2 = negative control, Lane 3–5 = transgenic line TRV-vAA T2-1, Lane 6–8 = transgenic line TRV-vAA T2-3 and Lane 9–11 = TRV-vAA T2-6 (**B**) Amplification of kanamycin gene the VDPS potato lines by PCR amplification of fragment size 195 bp. Loading sequence of the samples in 1.2% agarose gel is, M = 1 kb DNA Marker, Lane 1 = positive control, Lane 2 = negative control, Lane 3–5 = transgenic line TRV-*AChE* T1-3, Lane 6–8 = transgenic line TRV-*AChE* T1-7 and Lane 9–11 = TRV-*AChE* T1-8 (**C**) Amplification of kanamycin gene the VDPS potato lines by RT-PCR-amplification of fragment size 154 bp. Loading sequence of the samples in 1.2% agarose gel is, M = 1 kb DNA Marker, Lane 1 = positive control, Lane 2 = negative control, Lane 3–5 = transgenic line TRV-*EcR* T2-3, Lane 6–8 = transgenic line TRV-*EcR* T2-4 and Lane 9–11 = TRV-*EcR* T2-8.

**Figure 6:**
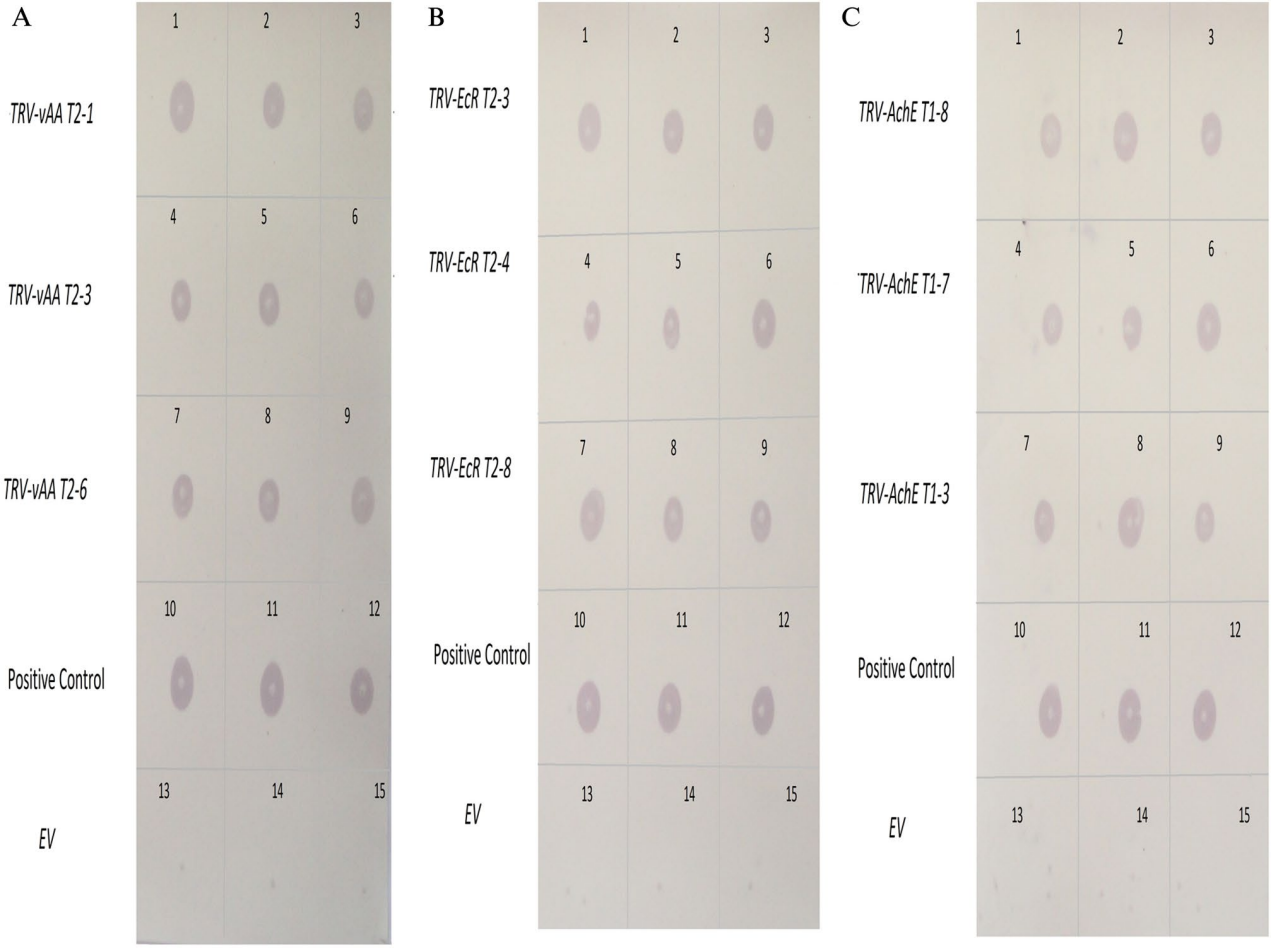
RNA dot blot showing transcripts of insect’s genetic target in VDPS potato lines (**A**) Confirmation of the *v-ATPaes-A* transcripts in the VDPS potato lines: Block 1–3 = transgenic line *TRV-vAA* T2-1, Block 4–6 = *TRV-vAA* T2-3, Block 7–9 = *TRV-vAA* T2-6, Block 10–12 = positive control and Block 13–15 = control plants (*TRV-EV*); (**B**) Confirmation of the *EcR* transcripts in the VDPS potato lines: Block 1–3 = transgenic line *TRV-EcR* T2-3, Block 4–6 = *TRV-EcR* T2-4, Block 7–9 = *TRV-EcR* T2-8, Block 10–12 = positive control and Block 13–15 = control plants (*TRV-EV*) (**C**) Confirmation of the *AChE* transcripts in the VDPS potato lines: Block 1–3 = transgenic line *TRV-AChE* T1-8, Block 4–6 = *TRV-AChE* T1-7, Block 7–9 = *TRV-AChE* T1-3, Block 10–12 = positive control and Block 12–15 = control plants (*TRV-EV*).

Because the dsRNA expressed by bacteria caused significant mortality, the same fragments for the three candidate genes were selected for expression in potato plants. Gene fragments were cloned in the VIGS vector *pTV* in an antisense orientation. *Agrobacterium*-mediated transformation was carried out to insert the fragments into the potato plants. After transformation, transgenic plants were confirmed by PCR analysis (Fig. 5A–C). The expression of exogenous genes in VDPS plants was also confirmed by dot blot analysis using appropriate probes. For all three VDPS experimental plant lines, negative control plants (EVs) did not show any of the target RNAs (Fig. 6A–C).

**Figure 7:**
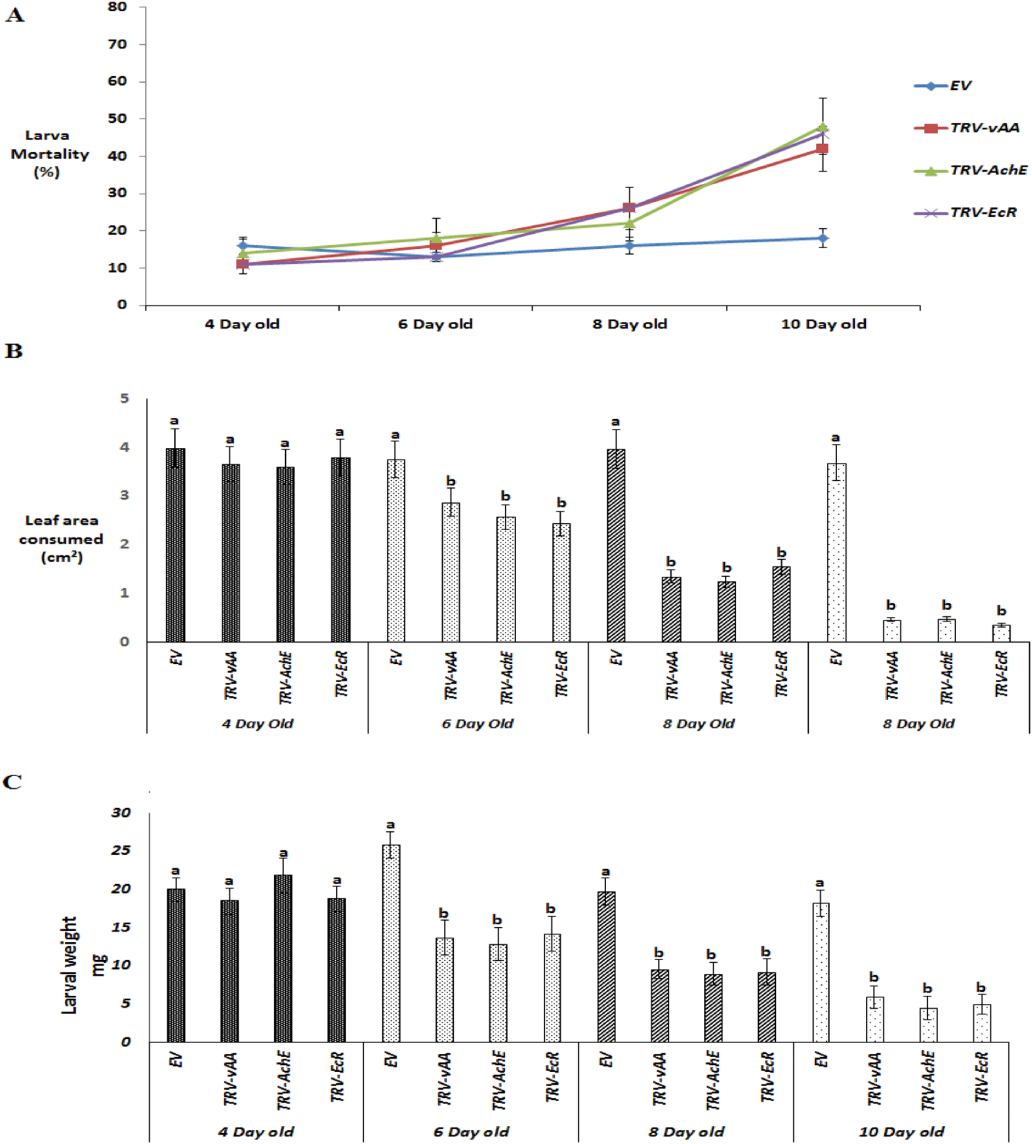
*In-vivo* Feeding bioassay on VPDS plant. Mortality (**A**) of 1st instar *H. armigera* larva after 5 days of feeding on 4, 6, 8 and 10-day-old transformed plants. All data are means ± SD (*n* = 30). Leaf area consumed (**B**) by *H. armigera* larva. Average larval weight (**C**) of *H. armigera* larva after 5 days of feeding on 4-, 6-, 8- and 10-day-old transformed plants. *TRV-EV* was used as a control plant. For each experiment 30 larvae were used and p < 0.05 was considered as significant.

The age of the plants used in this trans-kingdom RNAi study varied greatly. A preliminary in-vivo experiment on a VDPS potato plant revealed that the plant after 10 days of infiltration with the *TRV-VIGS* system was toxic to *H. armigera* larva compared to the control plant transformed with empty *TRV* vector. Leaf area consumed and larval weight gain on plants of different ages (4, 6, 8, and 10 dpi) were evaluated. After 5 days of feeding on the plants, ≥ 80% of the larva succeeded in surviving from 4 days post infestation and 6 days post infestation plants. It was observed that the larva fed for 5 days on 10-day-old VDPS plants expressing *ds- EcR* failed to gain much body weight compared to the control plants of the same age (Fig. 7C). The larval survival rate was dropped by approximately 55% in each group (Fig. 7A). However, the ingestion of leaves from 10-day-old VDPS plants expressing *ds-AChE* was minimal (Fig. 7B), and stunned larval growth and significant mortality were recorded compared to the control plants. Therefore, all in-vivo feeding bioassays on transformed plants were performed with 10 dpi plants.

### Transgenic (transient) potato plants expressing dsRNA show enhanced resistance to *H. armigera*

**Figure 8:**
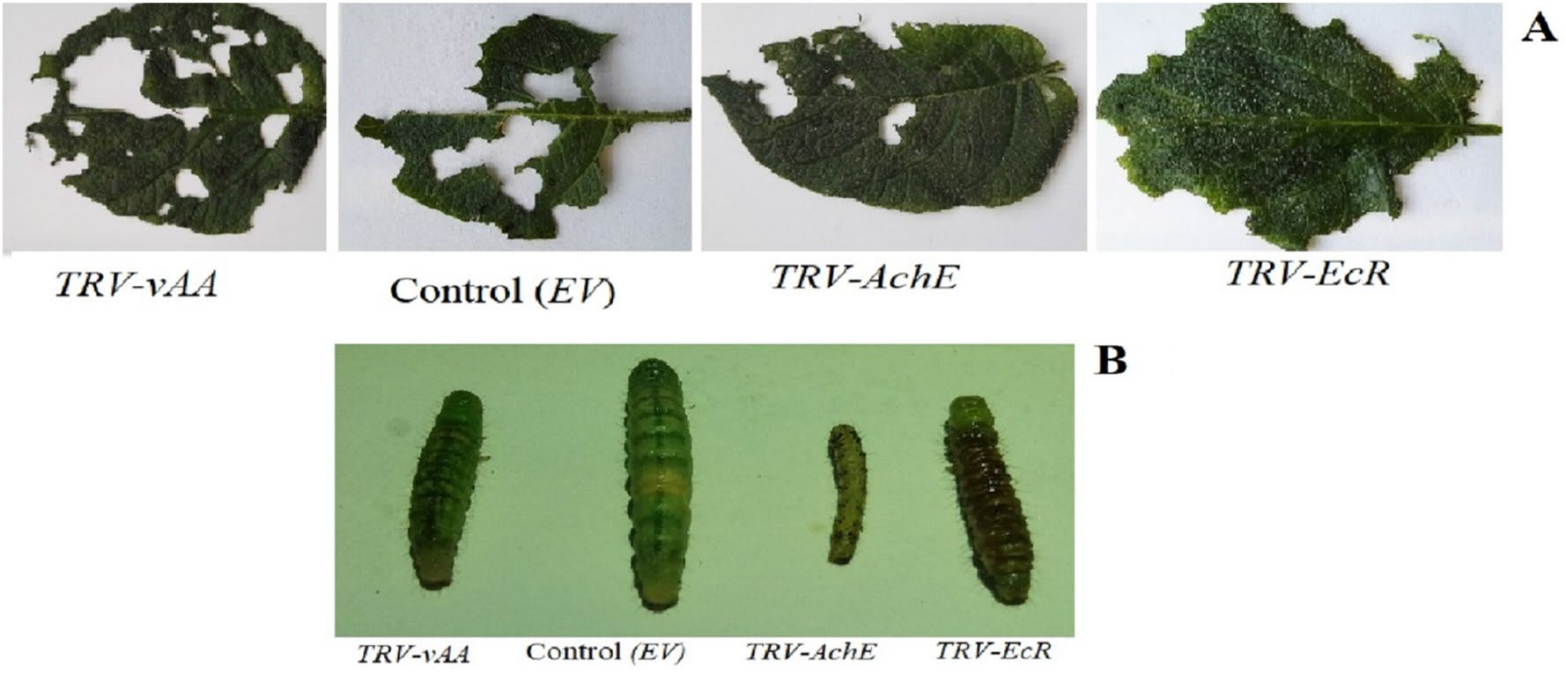
*H. armigera* feeding assay on VDPS plants. *In-vivo* feeding assay with 10-day-old transformed plants. Detached leaves of transformed plants are represented in (**A**) *H. armigera* body size (**B**) surviving after 10 days of feeding on *TRV-EV* (Control)*, TRV-AChE, TRV-vAA* and *TRV- EcR* potato plants.

**Figure 9:**
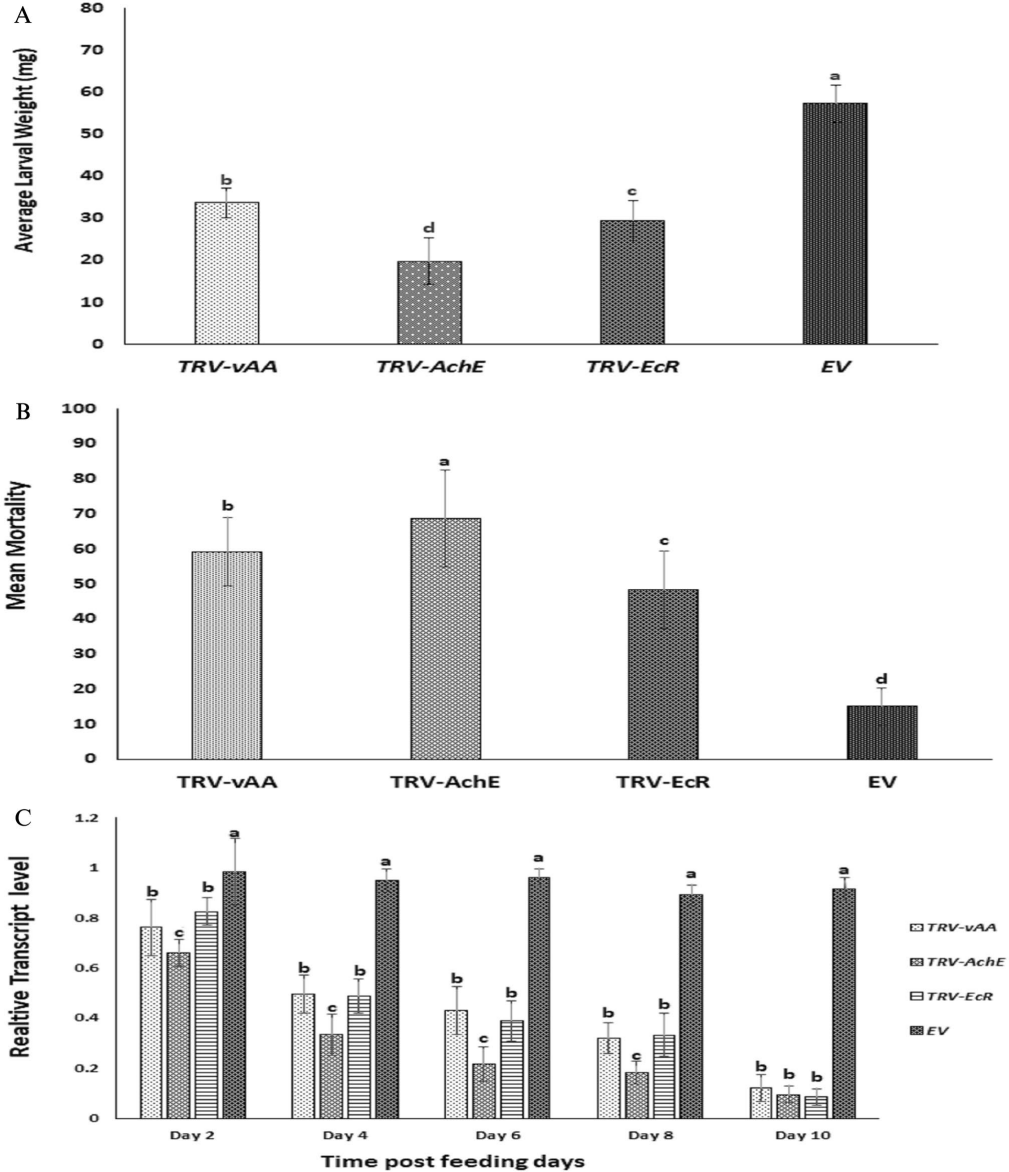
Average larval weight (**A**) mortality (**B**) and relative gene expression (**C**) of *H. armigera* 1st instar larva post feeding exposure on VDPS potato lines compared with the control presented in a bar chart. All data are the means ± SD (n = 30) after 10 days of larval feeding on 10-day-old transformed and control plants. Significant differences from the control were identified by analysis of variance using ANOVA. Different letters above standard deviation bars indicate significant differences. All experiments were triplicated. p < 0.05 was considered as significant.

To evaluate whether VDPS potato plants expressing *ds-AChE, ds-EcR* and *ds-vATPase-A* exhibited adverse effects on *H. armiger*a, newborn larvae were allowed to feed on potato plants. After 10 days of feeding, it was evident that plants transiently expressing the target genes dsRNA, especially *AChE*, showed a much greater resistance to *H. armigera* than the control plants. The leaves of *TRV-AChE* were damaged significantly less than those of the control plants. Compared with the other transformed plants, larva fed on *TRV-vAA* potato plants consumed more leaves than those fed on *TRV- EcR* and *TRV-AChE* plants (Fig. 8A). The body weight gain of larva fed on *TRV-AChE, TRV-EcR* and *TRV-vAA* plants was found to be significantly less than that of the control plants. Additionally, the first instar larva that were fed on whole potato plants exhibited a greater difference in body weight. It was apparent that the leaves of transgenic potato plants expressing *AChE* dsRNA exhibited much greater resistance to *H. armigera* than those of control plants after 10 days (Fig. 8A). VDPS plants expressing *vATPase-A* and *EcR* dsRNA show somewhat lower resistance than *TRV-AChE plants*. Abnormal weight gain and growth in larva fed on VDPS plants became apparent by day 7, most notably the *TRV-AChE* lines. The body size and body weight of larva fed on the *TRV-EcR* and *TRV-vAA* lines were also significantly reduced compared to those of the control group (Figs. 8B, 9A).

Moreover, bioassays on the transiently transformed plants caused a significantly higher mortality rate than the control plants. The larval survival rate was much higher in the control group than in the experimental group. However, highly significant variation was found between the experimental and control groups. After 10 days of feeding, larval mortality in the experimental groups ranged from 48 to 68%, which was significantly higher than the 17% recorded in the control group (p < 0.05). Among the larva that were fed on the transgenic potato plants, those on *TRV-AChE* leaves had higher mortality (68%) than those fed on *TRV-EcR* (48%) and *TRV-vAAA* (59%) lines (Fig. 9B). Insect bioassay experiments verified that transgenic plants transiently expressing dsRNA targeting *AChE, EcR and vAA* could harmfully affect *H. armigera* growth and survival.

### Consuming transgenic plants suppressed gene expression in *H. armigera*

To confirm that the transgenic plants induced gene silencing in the insects, the mRNA levels of each of the target genes were quantified using qRT-PCR. In accordance with the above mentioned in-vivo feeding bioassay, the larva released on the *TRV-EcR, TRV-AChE and TRV-vAA* transgenic lines were used for qPCR analysis, and time-dependent analysis of all three targeted mRNAs was carried out. In the larva fed on *TRV-EcR* plants, the mRNA level remained mostly unaffected on day 2 and began to decrease on day 4 (53%). In larva feeding on *TRV-AChE* plants, *AChE* transcript levels dropped by 63% on day 2 compared with those reared on control plants. Significantly reduced levels of endogenous *vAA* expression (88%) were detected in the larva reared on *TRV-vAA* plants on day 10 (Fig. 9C).

## Discussion

Sequence-specific knockdown of mRNA transcripts by RNAi provides countless opportunities for crop protection, such as pest control and the reduction of crop damage by invading pathogens. An appropriate delivery strategy for dsRNAs can be a foremost restraint of RNAi studies for pests^29–31^. In this study, we demonstrated the induction of sequence-specific RNAi effects in *H. armigera* by consumption of artificial diets coated with dsRNA and VDPS potato plants.

To improve the efficacy of RNAi, an efficient approach for dsRNA delivery is required. Moreover, stabilization of dsRNA in the body of targeted insects (gut or hemocoel) is the key feature of an effective dsRNA delivery method. There are chances of dsRNA degradation by nucleases in the gut lumen and some tissues of insects^32^. For instance, dsRNase found in the saliva of *Lygus lineolaris* has been reported to catalyze specific cleavage of dsRNA^33^. Similarly, dsRNA degradation was also recorded in the pea aphid *Acyrthosiphon pisum* after oral delivery of an artificial diet coated with dsRNA^34^. A specific dsRNase in the midgut of *Bombyx mori* has been reported to play a central role in the degradation of RNA and DNA, primarily to protect against viral attack by digestion of viral nucleic acids^35^. Four different types of dsRNase have been reported in *Schistocerca gregaria*, and the RNAi efficacy of dsRNAs can be improved by targeting dsRNase 2^36^. These reports proposed that the body fluid of *H. armigera* might have dsRNase activity. dsRNase activities relied on the larval developmental stages and tissues. dsRNA targeting the ultraspiracle gene of *H. armigera* has revealed rapid digestion in midgut juice compared to hemolymph^37^. These findings reveal that high dose of dsRNA or continuous oral delivery would be required to achieve effective knockdown.

The desired results of RNAi experiment varies with the dsRNA dose, insect life stage, species, transcript abundance of genetic target and delivery approaches^38^. To determine the optimum dose of dsRNA in our study, different concentrations of bacterially expressed dsRNAs were mixed with artificial diet and fed to newly hatched caterpillars. Increased doses significantly increased RNAi efficacy and insecticidal activity. Moreover, the findings of this study revealed that the optimal target developmental stage *of H. armigera* was newly hatched larva, for which it causes 60% mortality. Such lethal activity of dsRNA is higher than the findings of other studies conducted on lepidopteran insects. For example, targeting the *β integrin* subunit of *S. exigua* through bacteria expressing dsRNA caused only 50% mortality^39^. RNAi against the immunosuppressive chymotrypsin gene of *S. exigua* resulted in only 45% mortality even at higher concentrations of dsRNA^40^. RNAi efficiency was enhanced with increased doses of dsRNA in the cotton bollworm *Pectinophora gossypiella*^41^. In another study conducted on *H. armigera*, relatively stable RNAi efficacy and high mortality was detected when high dose of dsRNA application was used^42^. The deceased survival rate of larva revealed by this study might be due to the continuous oral delivery of dsRNAs for prolonged timespan compared to the abovementioned study in which larvae were fed once or with low dsRNA concentration.

Quantitative RT-OCR data showed that dsRNA coated artificial diet triggered significant depletion of mRNA levels in *H. armigera* compared to the control group. A maximum 89% decrease was detected on day 4 for the *EcR* group, 91% decrease for *vAA* on day 4, and 94% decrease for *AChE* on day 10 through oral intake of each dsRNA. Our results were slightly different from those of a previous study using oral consumption of dsRNA. Our quantitative RT-PCR data proposed that oral delivery to *H. armigera* could accomplish similar knockdown effects as in other insects. For example, oral delivery of dsRNA to the termite *Reticulitermes flavipes* reduced the relative mRNA expression of *Cell-1* and *Hex-2 by* approximately 60% on day 2^43,44^. Furthermore, a significant deasecre (42%) in *salivary gland nitrophorin 2* was demonstrated 48 h after ingesting dsRNA in *Rhodnius prolixus* (45). A study on *E. postvittana* and *S. frugiperda* also revealed that ingesting dsRNA could cause depletion of transcript levels in various pest orders^45,46^.

Based on the RNAi effect at the transcript level, we assumed a high mortality rate in the *v-ATPase-A, EcR* and *AChE*-fed groups compared to the control *egfp* group. Moreover, higher mortality was also observed in a dsRNA feeding assay targeting the *v-ATPase-A* gene of Western Cotton Rootworm larva^13^. Another study also demonstrated the usefulness of targeting the ATPase-A gene to control other insect species^47^. In our findings, the mortality of *v-ATPase-A* was relatively high (72%) compared to the same target in a study conducted on adult *Bactrocera dorsalis*^48^. Additionally, a lower mortality rate was observed in the *ds-EcR* and *ds-AChE* test groups even after 10 days of feeding than in a recent study conducted by Malik et al*.* on white flies^49^. There might be several reasons underwrite this phenomenon. First, larvae were used as the object of study compared to adult flies used in the study conducted by Li et al*.*^48^. Similar results were demonstrated by Baum et al. for the *WCR* feeding assay^10^.

Several studies established successful insect gene knockdown with *Agrobacterium*-mediated transient or stable transgenic plant-derived RNAi^21,50^. We report parallel gene silencing for *AChE, EcR* and *vAA,* demonstrating that TK-RNAi is a reproducible and robust approach to control harmful insect pests. Trans Kingdom RNAi (TK-RNAi) has the potential to be a crop protection tool targeting harmful insects with greater efficacy than presently existing pesticides or transgenic crops expressing *Bt toxin*^51^. Particularly in the case of a lepidopteran insect, *AChE, EcR* and *vAA,* which are involved in neural signaling, the steroid signaling pathway and hydrolyses of ATP, respectively, are potential genes to be targeted through RNAi. Silencing reduces the transcript level of the target gene, reducing its activity and influencing larval development and mortality^29^. Therefore, in this first attempt at insect gene knockdown by a plant virus-based dsRNA-producing system, we considered *AChE, EcR* and *vAA* to be vital candidate genes to control *H. armigera*. TK-RNAi-mediated gene silencing in the present study involving the same targeted genes showed mortality rates of approximately 68% and 48% in *TRV-AChE* and *TRV- EcR,* respectively. Our findings were somewhat different compared to a study conducted by Malik et al.,^49^ which demonstrated that transcript levels of *AChE* and *EcR* were down-regulated significantly in White flies in response to dsRNA-producing *N. benthamiana*, resulting in a higher level of mortality even after five days.

Successful TK-RNAi in *H. armigera* permitted us to employ it as a standard for developing a faster and easier VDPS approach. Since VDPS is a robust approach, we could evaluate three genes in a short time and found *AChE* to be a vital target for pest control. Since larva failed to gain weight and body size was significantly reduced feeding on TRV-*AChE* potato plants compared to control plants (Figs. 8B, 9A), this gene among the three may play a key role in *H. armigera* resistance to plant ingestion. However, using VDPS, which was recently effective in root nematodes, we demonstrated that VDPS- and bacterial-expressed dsRNA-mediated gene silencing was somewhat similar. Nevertheless, somewhat different mortality rates were observed in the two distinct approaches. Larva fed *TRV-vAA* and *TRV-AChE* showed reduced mortality rates compared with those fed a dsRNA-coated diet. Moreover, larva reared in the *TRV-vAA* group showed an increased mortality rate than those that ingested a diet coated with bacterial-expressed dsRNA (Figs. 4A, 9B). Data obtained from the *EcR* groups fed either a dsRNA-coated diet or transformed plants showed similar molting abnormalities as larval-pupal intermediates in *H. armigera.* (Fig. 4B) showing a lethal phenotype comparable to *B. mori*^52^. However, neither larval treatment groups of *AChE* and *v-ATPase-A* involving dsRNA-coated diet nor plant-mediated RNAi showed abnormal development.

Parallel to PMRI, the silencing efficacy of VDPS was also demonstrated to be highly precise that was previously notable against root nematodes and *M. sexta*^53–55^ for all three target genes. PMRi is obviously the approach of choice for crop resistance in geographical areas that permits the use of GMO crops and could be of immediate usefulness in the management of devastating insects such as *H. armigera*. Nevertheless, VDPS could be used as a productive approach for high-throughput RNAi screening of potential genes in insects. The size of dsRNA is the main concern in RNAi studies. Most of the studies on pests involved oral intake of dsRNA, and a 300–520 bp fragment size has been used^56^. In TK-RNAi against nematodes involving VDPS, fragments larger than 150 bp were endorsed^53^. However, integration of small sense or antisense inserts, mainly small hairpins, was demonstrated to be productive involving TRV-based gene silencing^57^. Our results were aligned with the findings in *M. sexta*^55^ and are consistent with the supposition that the effectiveness of RNAi is contingent on the size of the consumed dsRNA.

More notably, the present study established the usefulness of the RNAi mechanism for precise and robust knockdown of genetic elements in lepidopteran insects evolving resistance to transgenic *Bt* plants. This approach demonstrates a new method of insect management that might aid presently used *Bt*-expressing transgenic plants^58^. Discrete genomic and physiological changes are anticipated to occur between agriculturally harmful pest species, making the vigilant selection of genetic targets(s) for specific insect species fundamental while also considering the adverse effects on non-target or beneficial insects that could encounter transgenic plants. Several considerations are thereby vital for the achievement of harmless and effective trans-kingdom RNAi-mediated insect control. However, recalling all the potential of TK-RNAi, VDPS proved to be a robust and resourceful substitute appropriate for ecological investigation.

## Conclusions

In summary, optimization of oral delivery of bacterial expressed dsRNA to control the lepidopteran pest *H. armigera* was made*.* Finally, dsRNA concentrations ≥ 15 µg/day/larva was found to be able to reduce larval survival and growth. Findings of present study demonstrate that targeting *acetylcholinesterase* (*AChE*),* ecdysone receptor *(*EcR*) and *v-ATPase-A *(*vAA*) genes was effective to control population, growth and development of *H. armigera.* The perseverance of the silencing efficacy also requires further study due to the high survival rate of the 5th instar larvae. Moreover, TK-RNAi, In addition, the currently available data suggests that insect-resistant transgenic plants generated using VDPS mediated silencing of the insect gene may also represent useful and alternate strategy for insect pest management.

## Supplementary Information

Supplementary Information 1.

Supplementary Information 2.

Supplementary Information 3.
