# Supplementary material for: Silencing of multiple target genes via ingestion of dsRNA and PMRi affects development and survival in Helicoverpa armigera
Source: Sci Rep. 2022 Jun 21;12:10405. doi: 10.1038/s41598-022-14667-z (PMC9213516; doi:10.1038/s41598-022-14667-z)
Supplement: Supplementary file 1 — Supplementary Information 1. [file 41598_2022_14667_MOESM1_ESM.docx]

**Original Gel figures**

**Silencing of multiple target genes via ingestion of dsRNA and PMRi affects development and survival in *Helicoverpa armigera***

**Muhammad Nauman Sharif^1*^, Muhammad Shahzad Iqbal^2^, Rukkaya Alam^3^, Mudassar Fareed Awan^4^, Rao Muhammad Tariq^1^, Qurban Ali^1,5*^and Idrees Ahmad Nasir^1^**

1. Center of Excellence in Molecular Biology, University of the Punjab, Lahore, Pakistan

2. Department of Biotechnology, Faculty of Life Sciences, University of Central Punjab, Lahore, Pakistan

3. Department of Zoology, University of the Punjab, Lahore, Pakistan

4. University of Management and Technology, Sialkot, Pakistan

5. Institute of Molecular Biology Biotechnology, The University of Lahore, Lahore, Pakistan.

Corresponding Author Email address: [nauman.cemb@gmail.com](mailto:nauman.cemb@gmail.com), [saim169@gmail.com](mailto:saim169@gmail.com)


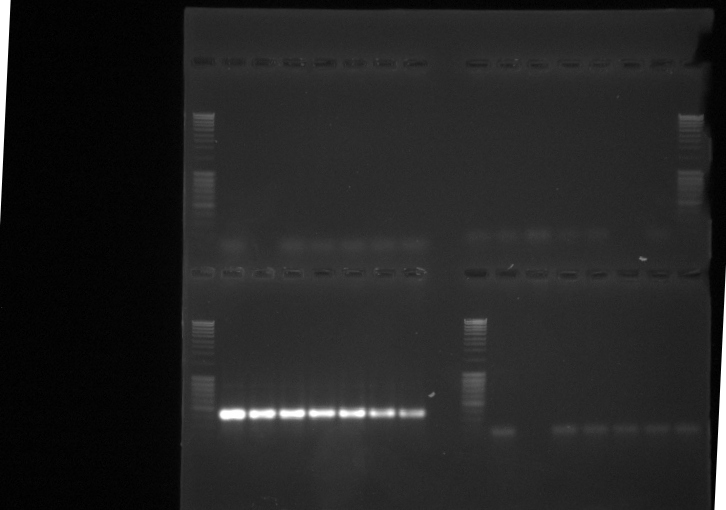


**Figure 1. Stability of dsRNA on artificial diet**. dsRNA from artificial diet was extracted and examined by agarose gel electrophoresis for integrity at different intervals of time.


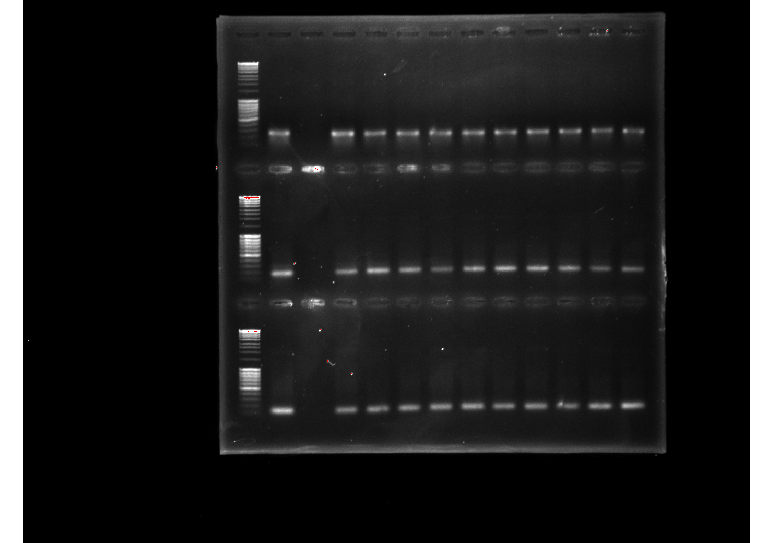


**Figure 5. Molecular analysis of VDPS plants.** (**A**) Amplification of the kanamycin gene in the TRV-vAA potato lines by PCR amplification of fragment size 251 bp. Loading sequence of the samples in 1.2% agarose gel is, M = 1 kb DNA Marker, Lane 1 = positive control, Lane 2 = negative control, Lane 3 –5 = transgenic line TRV-vAA T2-1, Lane 6–8 = transgenic line TRV-vAA T2-3 and Lane 9-11 = TRV-vAA T2-6 (**B**) Amplification of kanamycin gene the VDPS potato lines by PCR amplification of fragment size 195 bp. Loading sequence of the samples in 1.2% agarose gel is, M = 1 kb DNA Marker, Lane 1 = positive control, Lane 2 = negative control, Lane 3 –5 = transgenic line TRV-*AChE* T1-3, Lane 6–8 = transgenic line TRV-*AChE* T1-7 and Lane 9-11 = TRV-*AChE* T1-8 (**C**) Amplification of kanamycin gene the VDPS potato lines by RT–PCR-amplification of fragment size 154 bp. Loading sequence of the samples in 1.2% agarose gel is, M = 1 kb DNA Marker, Lane 1 = positive control, Lane 2 = negative control, Lane 3 –5 = transgenic line TRV- *EcR* T2-3, Lane 6–8 = transgenic line TRV- *EcR* T2-4 and Lane 9-11 = TRV- *EcR* T2-8
