# Supplementary material for: Silencing of multiple target genes via ingestion of dsRNA and PMRi affects development and survival in Helicoverpa armigera
Source: Sci Rep. 2022 Jun 21;12:10405. doi: 10.1038/s41598-022-14667-z (PMC9213516; doi:10.1038/s41598-022-14667-z)
Supplement: Supplementary file 2 — Supplementary Information 2. [file 41598_2022_14667_MOESM2_ESM.pdf]

Supplementary Material

Silencing of multiple Target Genes via ingestion of dsRNA and PMRi affects development and survival in *Helicoverpa armigera*

Muhammad Nauman Sharif[1], Muhammad Shahzad Iqbal[2], Rukkaya Alam[3], Mudassar Fareed Awan[4], Rao Muhammad Tariq[1], Qurban Ali[5*] and Idrees Ahmad Nasir[1]

1. Center of Excellence in Molecular Biology, University of the Punjab, Lahore, Pakistan.
2. Department of Biotechnology, Faculty of Life Sciences, University of Central Punjab, Lahore.
3. Department of Zoology, University of the Punjab, Lahore, Pakistan.
4. University of Management and Technology, Sialkot, Pakistan
5. Institute of Molecular Biology Biotechnology, The University of Lahore, Lahore, Pakistan.

Corresponding Author Email address: nauman.cemb@gmail.com, saim169@gmail.com

Primers used

Primer Name	Sequence 5′ to 3′	Purpose
AchE-F	TTCAAACGAGGCATCTTGC	Amplification
AchE-R	TTGCCAGTCAAAGCGAATC	Amplification
EcR-F	TCCAGCTTCAAGTGTGAACG	Amplification
EcR-R	CATATCCGAGTCCTCGTCGT	Amplification
vAA-F	TATCCTGGGCTCCATCTTTG	Amplification
vAA-R	GACAGGCATGTTGGATGTGT	Amplification
AT-F	TCTCAATGCACCTGGCAGTA	Amplification
AT-R	AACGAGATGCTGAACAACCC	Amplification
AS-F	GAAATCAAGCGCGTACAACG	Amplification
AS-R	CCAGTGCTACTTCAACCCCA	Amplification
L4AT-F	GGCAAGCTTGCGTGACAGACCACACACTA	L4440 cloning
L4AT-R	GGCGAGCTCGGGTTGTTCAGCATCTCGTTC	L4440 cloning
L4AS-F	GGCAAGCTTTCACAGTAAACGCTGCACCT	L4440 cloning
L4AS-R	GGCGAGCTCTGGGGTTGAAGTAGCACTGG	L4440 cloning
L4AchE-F	GGCAAGCTTCCGGACACAATGATGAAAGA	L4440 cloning
L4AchE-R	GGCGAGCTCCCCTGGTGTGGTATTGAAGG	L4440 cloning
L4EcR-F	GGCAAGCTTGGCAGCACGAAGAGCTATGT	L4440 cloning
L4EcR-R	GGCGAGCTCCCTTGCGATCAACGACTTCT	L4440 cloning
L4vAA-F	GGCAAGCTTTCAACGAGCTCACACAGTCC	L4440 cloning
L4vAA-R	GGCGAGCTCCACAATGACATCGGAGTTGG	L4440 cloning
L4egfp-F	GGCAAGCTTCACATGAAGCAGCACGACTT	L4440 cloning
L4egfp-R	GGCGAGCTCTGCTCAGGTAGTGGTTGTCG	L4440 cloning
TAchE-F	GGCAAGCTTCCGGACACAATGATGAAAGA	TRV cloning
TAchE-R	GGCGGATCCCCCTGGTGTGGTATTGAAGG	TRV cloning
TEcR-F	GGCAAGCTTGGCAGCACGAAGAGCTATGT	TRV cloning
TEcR-R	GGCGGATCCCCTTGCGATCAACGACTTCT	TRV cloning
TvAA-F	GGCAAGCTTTCAACGAGCTCACACAGTCC	TRV cloning

TvAA-R	GGCGGATCCCACAATGACATCGGAGTTGG	TRV cloning
PAchE-F	CGACTGAATCCGGTGAGAAT	Plant detection
PAchE-R	GGTTGCATTCGATTCCTGTT	Plant detection
PEcR-F	AAATGCTTGATGGTCGGAAG	Plant detection
PEcR-R	TCAGGTGCGACAATCTATGG	Plant detection
PvAA-F	GGTTGCATTCGATTCCTGTT	Plant detection
PvAA-R	ATTCAACGGGAAACGTCTTG	Plant detection
qAchE-F	ACGAGATTCGCTTTGACTGG	qRT-PCR
qAchE-R	ATCATTCCAGAAAGCGCAAG	qRT-PCR
qEcR-F	GGTGCTCGACTCACTCTTCC	qRT-PCR
qEcR-R	CTGACATCGGAGGTGCAGTA	qRT-PCR
qvAA-F	CTGACGTAGTGCTGGAGACG	qRT-PCR
qvAA-R	AGGGAAGAGTGAGTCGAGCA	qRT-PCR
qegfp-F	AGAACGGCATCAAGGTGAAC	qRT-PCR
qegfp-R	TGCTCAGGTAGTGGTTGTCG	qRT-PCR
q18s-F	GCCTCTTTGTCCAGATCAGC	qRT-PCR
q18s-R	ACTTCTTGGCTTTGGCAGAA	qRT-PCR
qβAct-F	TGCGTGACATCAAGGAGAAG	qRT-PCR
qβAct-R	TACCGATGGTGATGACCTGA	qRT-PCR

Sequences of cloned gene fragments.

v-ATPase-A

TCAACGAGCTCACACAGTCCATCTACATCCCCAAGGGTGTAAACGTACCTTCTCTGGCTAG
GGGTGTCAGCTGGGAATTCGTTCCCGTGAGTGTTAAGACGGGCTCCCACATCACTGGCGGA
GACCTGTACGGTCTGGTGCACGAGAACACGCTGGTGAAGCACCGCGTGTTGATCCCGCCCA
AGGCCAAGGGTACCGTCACATACATCGCGCCCGCTGGCAACTACAAAGTCACTGACGTAGT
GCTGGAGACGGAGTTCGACGGCGAGAAGGAGAAGTACACGGTGTTGCAGGTGTGGCCGGT
GCGTCAGCCGCGACCCGTCACCGAGAAGCTCCCCGCCAACCATCCGCTGCTCACCGGGCAG
AGGGTGCTCGACTCACTCTTCCCTTGCGTCCAGGGAGGTACCACTGCCATTCCCGGAGCTTT
CGGTTGCGGCAAGACTGTCATCTCGCAGGCGCTGTCCAAGTACTCCAACTCCGGTGTCATT
GTG

Ecdyson Receptor

GGCAGCACGAAGAGCTGTGTCTAGTCTGCGCCGACAGAGCCTCCGGATATCACTACAAAGC
ACTCACGTGTGAACGGTGTAAAGGTTTGTTCAGGCGGAGTGTAACCCAAAGTGCAGTGTAC
GTGTGCAAATTCGGCAGTGCTTGCGGAGTGGGTGTGTGTGCGGAGAAAGTGTCAGGAGT
GTAGGTTGAAGAAAGGTCTCGCGGTAGGCGTGAGGCCGGAGTGCGTGGTGCCGGAGAACC
AGTGTGCAGTGAAACGGAAAGAGGAAAAGGCGCAGAGGGAAAAGGACCAATTGCCCGTC

AGTACGACGACAGTAGACGATCACGTGCCTCCCATCGTGCAGTGTGATCCTCCGGGCCCAG
AGGCCGCTAGAATTCACGAGGTGGTGCCGCGGTTCCTCAGTGAGAAGCTCGTGGAACAGA
ACAGATTGAAGAAGGTGGCCCCCCTCACTGCCAATCAGAAGTCGTTGATCGCAAGG

Acetylcholine Esterase

CCGGACACAGTGGTGAAAGAAGGCAATTTCCATAATACCGAGGTGCTTCTTGGAAGTAATC
AAGACGAAGGAACCTATCTCTTACTATACGATTCCCTTGACTACTTCGAGAAAGACGGCAC
CAGCTCCTTGCAGCGGAAGCAAATTCTAGAAATTGTCGACACCATATTCAAGGATTTCTCC
AAAATAAAGAGCCAAGCTATCGTATTCCAATATACGGACTGGCAGGAAAATACCGGTGGC
TATCTGAACCAGGAAGTGATAGCTGACGTGGTGGGCGACTGTGTCTACGTGTGCCCTACTA
ACTACTTCGCAGAGGTGCTGGCCGATTCAGGAGTTGGTGTCCACTACTATTACTTCACACAT
CGCACCAGCACAAGTCTCTTGGGTGAGTGGATTGGCGTGGTGCAAGGCGGTGAGGTGGAGT
ACGTCTTCCGGCACCCGCTGAACGTGTTCCTTCAATACCACACCAGCG

egfp

CCACGTGAAGCAGCACGACTTCTTCAAGTCCGCCGTGCCCGAAGGCTACGTCCAGGAGCGC
ACCATCTTCTTCAAGGACGACGGCAACTACAAGACCCGCGCCGAGGTGAAGTTCGAGGGC
GACACCCTGGTGAACCGCATCGAGCTGAAGGGCATCGACTTCAAGGAGGACGGCAACATC
CTGGGGCACAAGCTGGAGTACAACTACAACAGCCACAACGTCTATATCGTGGCCGACAAG
CAGAAGAACGGCATCAAGGTGAACTTCAAGATCCGCCACAACATCGAGGACGGCAGCGTG
CAGCTCGCCGACCACTACCAGCAGAACACCCCCATCGGCGACGGCCCCGTGCTGCTGCCCG
ACAACCACTACCTGAGCA
